# The power of the feed-forward sweep

**DOI:** 10.2478/v10053-008-0022-3

**Published:** 2008-07-15

**Authors:** Rufin VanRullen

**Affiliations:** Centre de Recherche Cerveau et Cognition, CNRS, Université Toulouse 3, 31062 Toulouse Cedex 9, France.

**Keywords:** natural scenes, ultra-rapid categorization, pre-attentive recognition, backward masking, feed-forward processing, spike timing

## Abstract

Vision is fast and efficient. A novel natural scene can be categorized (e.g. does it contain an animal, a vehicle?) by human observers in less than 150 ms, and with minimal attentional resources. This ability still holds under strong backward masking conditions. In fact, with a stimulus onset asynchrony of about 30 ms (the time between the scene and mask onset), the first 30 ms of selective behavioral responses are essentially unaffected by the presence of the mask, suggesting that this type of “ultra-rapid” processing can rely on a sequence of swift feed-forward stages, in which the mask information never “catches up” with the scene information. Simulations show that the feed-forward propagation of the first wave of spikes generated at stimulus onset may indeed suffice for crude re-cognition or categorization. Scene awareness, however, may take significantly more time to develop, and probably requires feed-back processes. The main implication of these results for theories of masking is that pattern or metacontrast (backward) masking do not appear to bar the progression of visual information at a low level. These ideas bear interesting similarities to existing conceptualizations of priming and masking, such as Direct Parameter Specification or the Rapid Chase theory.

## Introduction

The hierarchical organization of the visual system (Felleman & Van Essen, 1991), and the presence in its higher levels of object- and category-selective neurons (Gross, Rocha-Miranda, & Bender, 1972; Logothetis, Pauls, & Poggio, 1995; Kreiman, Koch, & Fried, 2000; Perrett, Rolls, & Caan, 1982; Quiroga, Reddy, Kreiman, Koch, & Fried, 2005), suggest that a feed-forward wave of neuronal activation sweeping through the system may be sufficient to support rapid forms of recognition or categorization. On the other hand, the ubiquitous presence of anatomical feedback connections in the brain may imply a much more complex picture (Bullier, 2001). What exactly can be achieved by a single feed-forward sweep through the hierarchy, and by extension, what is feed-back necessary for? Here I summarize experimental and computational evidence showing that a feed-forward sweep can rapidly (in 150 ms or less) activate high-level category-selective representations, allowing for (at least a crude form of) object recognition or categorization; this ability does not depend on the availability of attentional resources (at least as long as the stimuli are spatially separated by an amount that prevents local competition within neuronal receptive fields); this feed-forward wave is (by definition) unaffected by backward masking, even at short stimulus onset asynchronies (SOA, 30 ms); it probably relies on no more than one or two spikes for each implicated neuron, suggesting a potential role for spike timing as a neuronal information carrier; it does not directly lead to conscious perception (conscious reports only become compatible with behavioral responses after a delay of about 100 ms).

## EARLY RECOGNITION IS RAPID BUT HIGH-LEVEL

**Figure 1.**
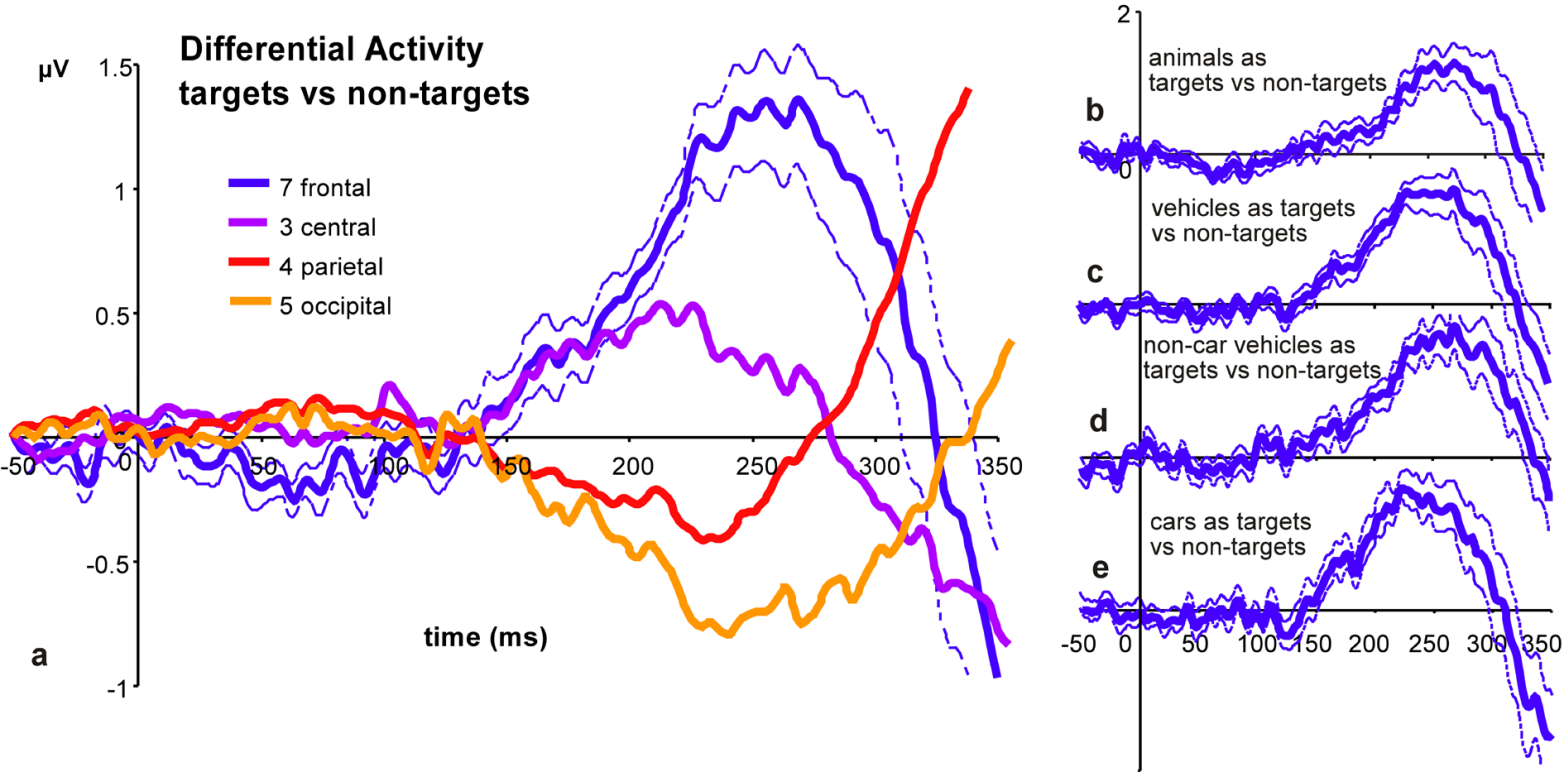
We recorded ERPs from 32 channels while 16 subjects categorized photographs of various types, e.g. animals, vehicles, landscapes, street scenes etc. On every other block, subjects were instructed to respond to pictures containing animals and to ignore all other pictures; on the remaining half of the blocks, subjects responded to pictures containing vehicles and ignored all others. For one given visual category, we could then compare (by computing a simple difference) the ERP signal generated by the photographs when they were treated as targets (and thus triggered a response) and when they were treated as non-targets (and had to be ignored). The comparison thus reflected the high-level, task-related status of the photographs, but not their physical properties (which were comparable in both cases). The resulting differential activity is shown on panel a for all visual categories averaged together, and for different electrode groups. Panels b-e represent the same comparison for the various categories: animals (a), vehicles (b) which were further separated into cars (e) and non-car vehicles (d). In each case, the difference is virtually zero up to about 150 ms, and diverges from zero after that time. This indicates that the decision of the subjects is reflected in the ERP after only 150 ms. Reprinted from VanRullen & Thorpe (2001).

How long does it take for the visual system to recognize or categorize a new object? A more physiologically oriented version of this question is, how long does it take to activate the corresponding object- or category-selective neurons in temporal cortex? If neuronal latencies in monkey IT are taken as an indicator, it seems that the answer would be about 100 ms or less (Keysers, Xiao, Foldiak, & Perrett, 2001; Oram & Perrett, 1992; Thorpe & Fabre-Thorpe, 2001; Vogels, 1999). ERPs in humans yield slightly higher estimates of 150 ms (Large, Kiss, & McMullen, 2004; Thorpe, Fize, & Marlot, 1996) to 170 ms (Bentin, Allison, Puce, Perez, & McCarthy, 1996; Jeffreys, 1996; Liu, Harris, & Kanwisher, 2002; Low, Bentin, Rockstroh, Silberman, Gomolla, Cohen et al., 2003), notwithstanding the occasional finding of more-than-ultra-rapid categorization in 50 ms or less (Mouchetant-Rostaing, Giard, Delpuech, Echallier, & Pernier, 2000; Seeck Michel, Mainwaring, Cosgrove, Blume, Ives et al., 1997). An important question is, of course, whether these early activations truly reflect an active categorization of the stimulus, or simply the unavoidable physical differences between the various image categories, which would show up in the ERP signals when hundreds of trials are averaged together. One of our experiments used an alternating dual-task to address this very question (VanRullen & Thorpe, 2001). We showed observers several hundred different scene photographs of various categories, including some containing animals, vehicles, landscape scenes etc. By asking subjects, on every other block, to respond to one given target category (say, animals) and ignore the others (including vehicles), and reverting these instructions on alternating blocks (respond to vehicles, ignore other scenes including animal scenes), we could isolate the processing related to the high-level status (target vs. non-target) of each category, all low-level differences being equated. For example, we could compare the ERP signals for animal photographs when they were treated as targets with the signals triggered by the same set of animal photographs when they were treated as non-targets. Results (reported in Figure 1) show a clear pattern of differential ERP activity starting up around 150 ms post-stimulus. This means that neuronal activity after this time can reflect the observer’s decision that a target is present on the screen, and not merely the physical properties of the photograph. In other words, the type of early recognition reflected in these signals is remarkably rapid, but can be considered a true high-level effect. Note that recent results (Kirchner & Thorpe, 2006) indicate that in a similar setting, but with two scenes presented on either side of fixation, saccadic responses to the side of a pre-specified target category (e.g. animal, vehicle) can be made much faster than any of the manual reports collected in the above-described experiments: the minimal selective saccadic reaction times can be as short as 120 ms, implying that the decision about the target location must have been taken in as little as 100 ms (counting at least 20 ms for the initiation of the saccadic response). The exact relation between this forced-choice paradigm and the pre-vious categorization tasks still needs to be worked out in more detail, but these new results clearly underscore the remarkable speed and efficiency of the visual system.

## EARLY RECOGNITION IS PRE-ATTENTIVE

Does rapid object recognition require attentional resources? Visual search, the gold standard of attentional paradigms (Treisman & Gelade, 1980) tells us that recognition or categorization are processes that simply cannot occur in parallel (Wolfe & Bennett, 1997). However, we have argued (VanRullen, Reddy, & Koch, 2004) that the question cannot be adequately addressed using the visual search paradigm, because the large size of object- and category-selective neuronal receptive fields will always prevent the (potentially pre-attentive) selective activation of these neurons when numerous stimuli are displayed simultaneously (which is, of course, the essence of the visual search paradigm). To get around this limitation, we have argued that one should focus instead on attentional manipulations that can take place with relatively isolated test stimuli. One example of such a paradigm is the dual-task paradigm (Braun & Julesz, 1998; Braun & Sagi, 1990): while attention is occupied by a difficult letter processing task at the center of the screen, one can test the ability of the subjects to recognize an isolated stimulus in the periphery. It turns out that under these conditions, photographs of animals, vehicles or faces can be categorized effortlessly, while apparently much simpler tasks (e.g. telling whether a vertically bisected colored disk is red-green or green-red) suffer dramatically (Fei-Fei, VanRullen, Koch, & Perona, 2005; Li, VanRullen, Koch, & Perona, 2002; Reddy, Reddy, & Koch, 2006; Reddy, Wilken, & Koch, 2004). The critical issue here seems to be that the task should involve natural or familiar semantic categories, rather than arbitrary categories (i.e. designed by the experimenter) that carry little meaning for the subject (Fei-Fei et al., 2005; VanRullen et al., 2004).

**Figure 2.**
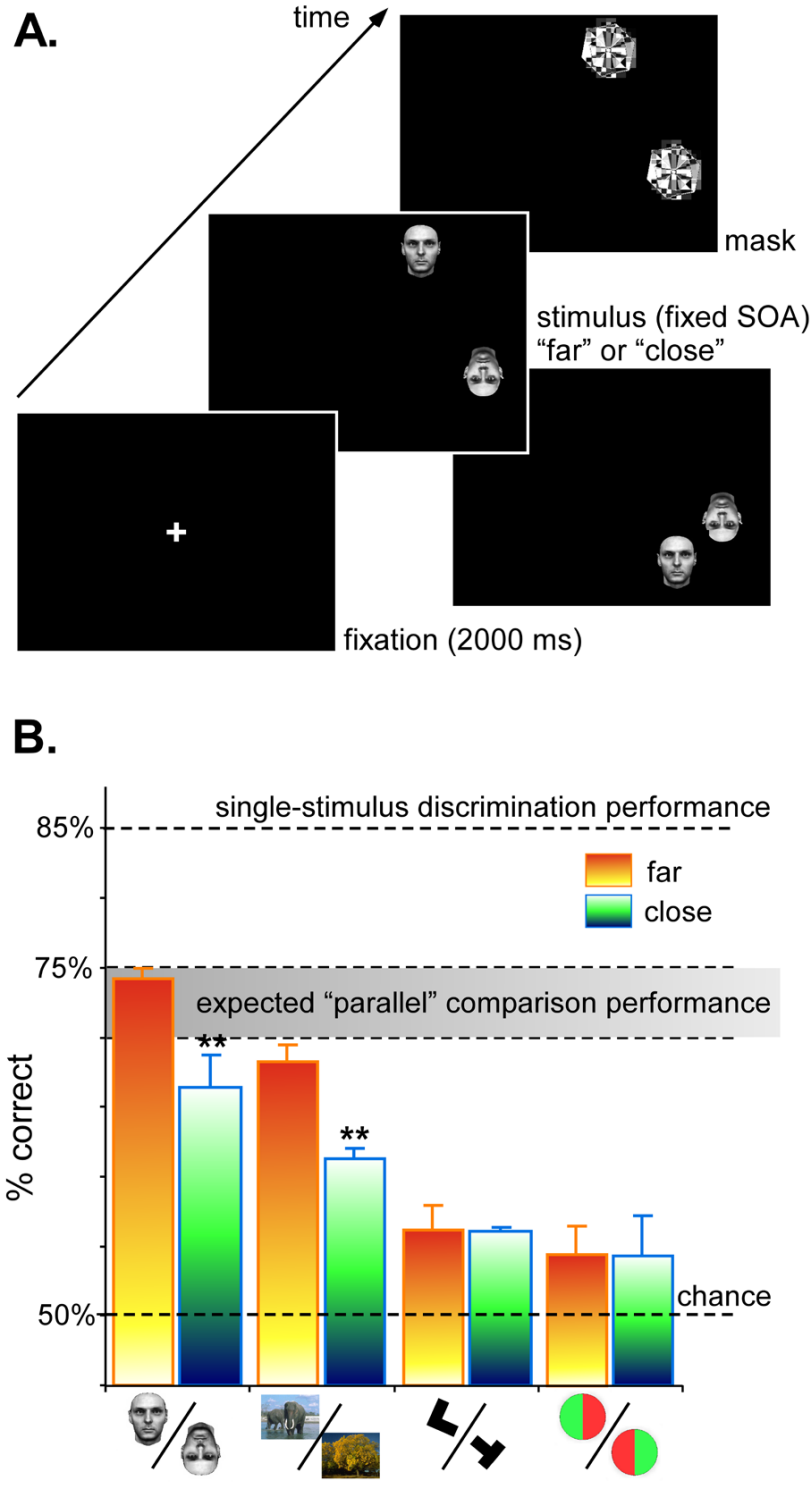
Comparison task. **A**. Two stimuli were shown at a time, at either 8º or 3º of spatial separation, and followed by a pattern mask. The SOA was adjusted for each subject and task so that each stimulus in isolation could be categorized at 85%. We tested whether the two simultaneous stimuli could be compared (i.e. a “same/different” category judgment) at the same SOA, as a function of the separation, for various categorization tasks: upright vs inverted faces, animal vs. non-animal scenes, randomly rotated L vs. T, bisected two-color disks. **B**. For the latter two tasks, comparison performance was very low (about 55%), and independent of the separation between items. For the two “natural” categorization tasks, comparison performance was near-optimal (between 70-75%) when the stimuli were far apart, but suffered significantly (down to 60-65%) when the spatial separation was decreased to 3º. This indicates that while artificial, arbitrary (i.e. experimenter-designed) stimulus categories always need attention to be processed, natural and familiar categories can be processed pre-attentively, as long as the local competition between stimuli is minimized. Reprinted from VanRullen et al. (2005).

More recently, we used a “comparison” paradigm to confirm and extend these results (VanRullen, Reddy, & Fei-Fei, 2005). Two stimuli were presented at the same time, followed by a mask. The SOA was adjusted so that it was possible to categorize each of the stimuli at 85% correct when presented alone. We tested whether subjects could compare the categories of the two simultaneously presented stimuli (i.e. “same/different” response), as a function of the spatial separation between them (Figure 2). This task required both stimuli to be correctly identified, since perfect identification of one of the two stimuli accompanied by guessing of the other one would still yield chance performance. We confirmed that artificial, experimenter-designed categories (e.g. bisected 2-color disks) could not be reliably compared under these conditions (whatever the distance between the objects), probably because attention is required for their processing. For natural image categories (animal vs. non-animal scenes, or upright vs. inverted faces), an interesting pattern emerged: comparison performance was near-optimal at the larger spatial separation (8º), confirming that the necessary processing can be done “in parallel”, i.e. without focused attention; but at the shorter spatial separation (3º), comparison performance was significantly decreased, suggesting that attentional demands were now more severe. We explained this effect in terms of competition between the stimuli within the large receptive fields of high-level cortical neurons: in our view, these neurons can be activated, even without attention, when an isolated stimulus is presented, and this activation underlies the rapid categorization effects described above; when two or more stimuli, however, fall into a single receptive field, competition prevents the selective activation of the neuron (Moran & Desimone, 1985; Reynolds & Desimone, 1999) and attention becomes necessary to resolve the conflict. In summary, the findings suggest that a pre-attentive, rapid sweep is sufficient to selectively activate high-level neurons in temporal cortex, provided that local competition between objects in the scene is minimal.

## EARLY RECOGNITION IS FEED-FORWARD

How can we test whether this rapid recognition abili-ty relies on a true feed-forward process? Backward masking is one straightforward way to address the question: in a system where early behavioral responses are determined by a pure feed-forward sweep, masking should not affect these early responses even at short SOAs. This is precisely what we found for an animal vs. non-animal scene categorization task: with a 30 ms SOA, the first 30 ms of correct behavioral responses were essentially unaffected by the presence of a mask (VanRullen & Koch, 2003). At the neurophysiological level, EEG investigations confirmed that, in the same animal vs. non-animal task, the backward mask does not annihilate the high-level target-specific response (Bacon-Mace, Mace, Fabre-Thorpe, & Thorpe, 2005). Instead, the amplitude of the response is directly proportional to the SOA.

**Figure 3.**
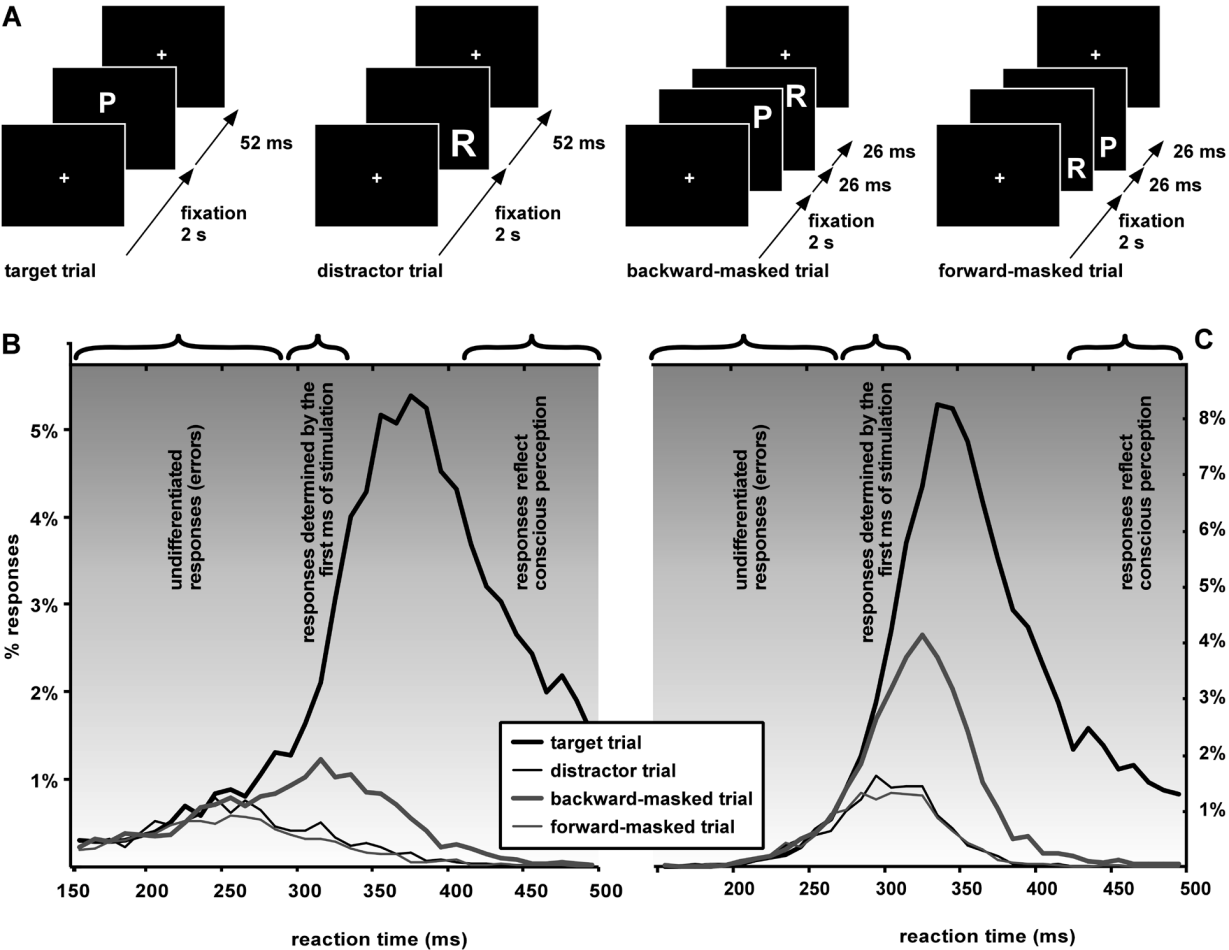
In a feed-forward system, the first selective behavioral responses to a target should be unaffected by the presence of a backward mask for a duration that is comparable to the SOA used. We tested this idea using a letter discrimination task. (**A**) Subjects were required to respond as fast as possible when the letter P was presented and withhold responding when the letters R or B were displayed (examples are shown here only with the distractor letter R). The letters’ location and size were randomized for each trial. In half of the trials, letters were flashed for 52 msec, while in the other half, two distinct letters were flashed successively for 26 msec (the target followed by a distractor in backward-masked trials, a distractor followed by the target in forward-masked trials). Under these conditions, due to backward and forward masking effects, only the distractor letter was consciously perceived. (**B**) Average distribution of RTs for 10 subjects (10-msec time bins). As predicted by the feed-forward model, responses to backward-masked trials followed the distribution of responses to targets for a certain period after the discrimination onset (290 msec). During this period, which lasted approximately 25 msec, behavioral responses were only determined by the first 26 msec of stimulation. After this period, the masking letter began to affect responses, but it was only after more than 415 msec that RTs fully reflected the subject’s perception of the stimulation. (**C**) Individual data for one additional subject who performed more than 42,000 trials. The discrimination onset for this subject was 275 msec, and the difference between targets and backward-masked trials appeared after 305 msec (i.e., 30 msec later). Backward-masked trials went down to the level of distractors after 435 msec. Reprinted from VanRullen & Koch (2003).

In our experiment (VanRullen & Koch, 2003), the pattern masks were designed to mimic the structure of natural scenes, with a 1/f Fourier power spectrum and a fine “wallpaper” texture superimposed. However, because such masks can only be expected to hide the relevant scene information “on average” (due to the large variability between the different photographs), it was difficult to assess whether the scene stimulus had been consciously perceived or not on every trial. Some local high-contrast scene information may have transpired through the mask on some trials. Thus, we investigated the same question using a set of more controlled stimuli (Fig. 3A): the target was now the letter P (size and screen position were randomized on every trial), and distractors were the letters B and R (display duration 52 ms for target or distractors); subjects were instructed to respond as fast as possible when the target was shown, but to refrain responding on distractor trials; on some “backward-masked” trials the target was shown briefly (for 26 ms) and followed by one of the distractors (for 26 ms as well) which served as a mask; on “forward-masked” trials one of the distractors now preceded the target letter, with the same display time (26 ms for each of the mask and target). The important aspect of this stimulus design was that, in virtually 100% of the masked trials (forward- and backward-masked), only the distractor letter component (i.e. the “mask”) was consciously registered (as assessed in a separate, non-speeded recognition session). On forward-masked trials, responses in the speeded categorization task were indistinguishable from the simple distractor trials, i.e. behavior and perception were compatible (Fig. 3B and 3C). But on backward-masked trials, we again found that the first 30 ms of correct behavioral responses reflected only the presence of the target letter, i.e. were unaffected by the presence of the backward mask – even though all that was consciously visible was the mask! This demonstrates that the early responses only depended on the first few milliseconds of the visual stimulation, and thus were probably triggered by a truly feed-forward sweep.

An important observation in our findings is the strong dissociation between motor responses and the subjective percept of our observers (in fact, the dissociation is complete for the earliest responses). This is directly compatible with classical observations of so-called “unconscious priming” (Ansorge, Klotz, & Neumann, 1998; Breitmeyer, Öğmen, & Chen, 2004; Jaśkowski, van der Lubbe, Schlotterbeck & Verleger, 2002; Jaśkowski, Skalska & Verleger, 2003; Neumann, 1990; Schmidt, 2002): fast motor responses can often reflect an unperceived prime rather than the following, consciously registered “mask”. In addition, however, we also found that the arrival of mask information within the system did not immediately erase the “prime” information, which appeared instead to linger in the system for an additional 100 ms (in other words, it took more than 100 ms for behavior to truly reflect the percept). This implies that access to conscious awareness cannot be directly granted by the feed-forward sweep, but that feed-back reentry on the order of 100 ms is required for awareness.

To recapitulate, the experimental findings presented thus far show that a feed-forward sweep through the visual cortical hierarchy rapidly activates high-level neurons selective to particular objects or categories. Even in the absence of attention, this activation is sufficient to support various forms of selective behavior (recognition, categorization), but apparently not to give rise to conscious perception.

## SURFING A SPIKE WAVE

Is it really possible to detect, recognize or categorize objects with a single pass of visual information through a hierarchy of areas using only feed-forward connections, and in a time compatible with the observed latency of high-level neurons? Simulations may allow us to assess the validity of this idea.

**Figure 4.**
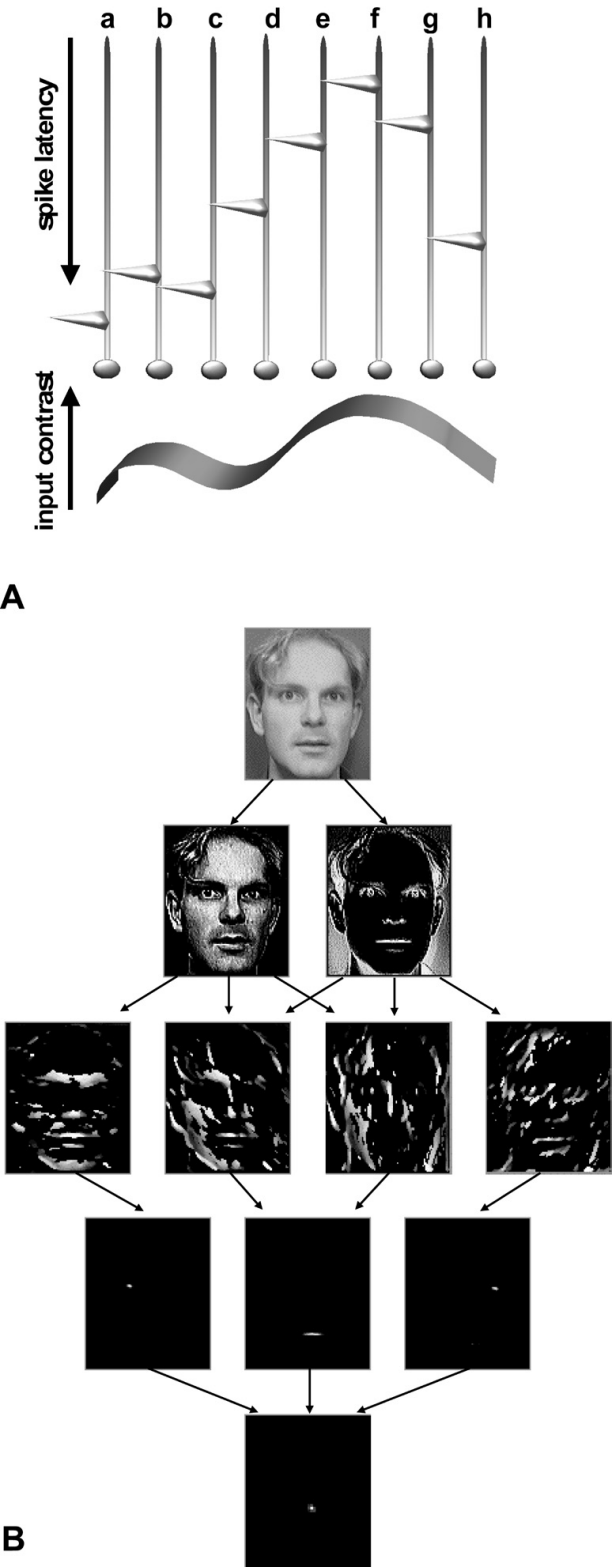
Simulations of the feed-forward propagation of a wave of spikes through a hierarchy of visual areas. **A**. Even when each neuron is only allowed to fire one spike, the pattern of firing order over a population can convey most of the stimulus information (the most activated neurons fire before the other ones). Using this scheme, it is possible to efficiently transmit visual information between two processing stages in 10-20ms, a time that is compatible with biological constraints. **B**. A photograph (top) is presented to a simple model with a 4-layer feed-forward architecture. Neurons in each of the 2 maps at the first level respond to local positive and negative contrasts in the input image. At the second level, neurons are selective to 8 different orientations (only 4 maps are shown here). At the next level, neurons were trained to respond to the firing pattern signalling the presence of a right eye, a mouth or a left eye. Finally, neurons in the last layer combine this information, and respond only when a face is present in their receptive field. This model can detect an arbitrary number of faces in natural photographs, with minimal numbers of false alarms, and in a time compatible with the speed of biological visual processing. Adapted from VanRullen & Thorpe (2002).

First, we must find a way of transmitting visual information in less than 10-20 ms per stage – to account for a firing latency of high-level neurons around 100 ms, and counting up to 10 synaptic stages separating the retina from high-level temporal cortex. During this time, most neurons will only have time to fire at most one spike (or up to two spikes for a small proportion of the neurons), so simply counting the spikes for each neuron would not seem to be an optimal strategy. Thus we decided to use the order in which neurons fire within a given population as the relevant variable (Gautrais & Thorpe, 1998; Thorpe, 1990). Indeed, the most activated neurons generally fire before less activated ones, and so the pattern of firing order over the population can reflect the amount of neuronal activation, even under conditions where each neuron only has time to fire one spike (Fig. 4A). This way, we can even limit (somewhat artificially) the number of spikes per neuron to a maximum of one, and then follow the propagation of this pure “first spike wave” throughout the system.

Second, we must choose an architecture that roughly reflects the hierarchical organization of the visual system. For example, for a face detection task, we used a 4-layer feed-forward organization (VanRullen, Gautrais, Delorme, & Thorpe, 1998): the first layer contained neurons selective to positive and negative local contrasts (corresponding roughly to retinal

ON-center and OFF-center ganglion cells); in the second layer neurons responded to local oriented edges at 8 different orientations (corresponding to V1 simple cells); neurons in the third layer detected the presence of certain facial features (e.g. left eye, right eye or mouth) in their receptive fields; finally, in the last layer, corresponding to IT cortex, neurons responded only to the correct combination of these facial features, i.e. to the presence of a face. In this simplistic model, the feed-forward connections between two layers were manually set to match the expected (i.e. the average) order corresponding to the to-be-detected property. In more recent studies (Guyonneau, VanRullen, & Thorpe, 2004, 2005) we have shown that this type of connectivity can also be “learned”, in a supervised or unsupervised manner, using a biologically plausible learning scheme based on spike time dependent plasticity (STDP).

As illustrated in Figure 4, this model was able to reliably detect the presence of a face in natural photographs, even when more than one face was presented at the same time in a reasonably cluttered scene. The higher-level, face-selective neurons virtually never responded to non-face photographs. The level of performance for this model was comparable to state-of-the-art face detection algorithms at the time (VanRullen et al., 1998). That a feed-forward architecture can support reasonably good recognition or ca-tegorization performance in natural scenes may not be fully surprising given the success of other related feed-forward models such as the Neocognitron (Fukushima & Miyake, 1982) or the HMAX model (Riesenhuber & Poggio, 1999). But ours remains the “only” model to date that can explain the extraordinary speed of the visual system, because it relies on the feed-forward propagation of the very first wave of spikes that are generated in the retina in response to scene onset. Using a similar design it is possible to perform efficient face detection (VanRullen et al., 1998), face identification (Delorme & Thorpe, 2001), and various other categorizations (Thorpe, Guyonneau, Guilbaud, Allegraud, & VanRullen, 2004). Without challenging the feed-forward nature of the network, the propagation of an asynchronous spike wave leaves considerable room for some refinements – including contour integration (VanRullen, Delorme, & Thorpe, 2001) or saliency-based processing (VanRullen, 2003). Most of this modelling effort is reviewed in (VanRullen & Thorpe, 2002).

## Discussion

I have shown electrophysiological and psychophysical evidence demonstrating that some forms of recognition or categorization, for object categories that are familiar and meaningful to the observer, can take place extremely rapidly, and with little attention – as long as local competition between objects is minimized. The finding that this rapid categorization is impervious to backward masking suggests that it must rely mainly on feed-forward mechanisms, and that it can be dissociated from conscious awareness of the stimuli, which apparently involves feed-back mechanisms. Computational simulations reveal that the feed-forward propagation of a single wave of spikes is indeed sufficient for at least a rudimentary form of recognition. How do these findings relate to other current theories of visual processing and, more specifically, masking?

The feed-forward sweep described here is very si-milar to the “transient channel” activation of Breitmeyer and colleagues’ dual-channel model (Öğmen, Breitmeyer, & Melvin, 2003) (see also Breitmeyer, this volume), in that it is able to activate the higher levels of the visual hierarchy, but does not directly determine the conscious visibility of a stimulus, which depends on later feed-back processes. This is also in agreement with the proposal by Moshe Bar that a fast but coarse, magnocellular-driven pass through the visual system can trigger a more selective top-down facilitation for the slower, parvocellular-driven object recognition processes (Bar, 2003; Bar, Kassam, Ghuman, Boshyan, Schmid, Dale et al., 2006). However, in our work we made no explicit assumption as to the parvocellular vs. magnocellular nature of early recognition: the feed-forward sweep may well affect both systems similarly, albeit at different times. Indeed, unconscious priming can also be observed for color stimuli, which primarily activate the parvocellular pathway (Breitmeyer et al., 2004; Schmidt, 2002).

Rapid and unconscious, yet selective behavioral responses are also a hallmark of theories based on so-called “unconscious priming”, such as the Direct Parameter Specification framework (Neumann, 1990; Jaśkowski, 1996; Ansorge et al., 1998) or the Rapid Chase model (Schmidt, 2002). Maybe the most important contribution of our work to these proposals could be the finding that this rapid unconscious processing can also extend to high-level categorization tasks involving complex natural stimuli.

Finally, our experimental results showing that rapid feed-forward recognition is also pre-attentive revives a speculation originally formulated by (Lamme, Super, & Spekreijse, 1998), who linked pre-attentive vision with feed-forward activity rather than with purely low-level processes:

“Pre-attentive and ‘early’ processing are intuitively associated with cortical areas low in the hierarchy. [However,] many feature conjunctions or complex stimulus attributes that are often encountered are probably engraved in the RF tuning properties of neurons in higher areas, such as the inferotemporal area. Instead of linking pre-attentive vision to primary cortical areas, it is probably best equated to feedforward, RF-based cortical processing.” (p. 533)

Later, Lamme and Roelfsema realized that local neuronal competition could potentially constitute a strong theoretical challenge for this identity between pre-attentive vision and feed-forward activity (Lamme & Roelfsema, 2000):

“It is therefore tempting to identify recurrent processing with attentive grouping. Pre-attentive processing, by contrast, could be identified with the feedforward sweep. This association appears to hold at a first approximation, but there are also several subtleties. First, most psychological theories suggest that attention is always required to group complex feature combinations. […] When elaborate feature constellations are embedded in a crowded search display, the feedforward sweep is curtailed. This is caused by inhibitory interactions among the representations of multiple objects, which are particularly pronounced at the higher hierarchical levels. Thus, the depth of pre-attentive encoding might depend on the number and spacing of display items.” (p. 576)

Our results can be viewed as a direct experimental confirmation of this proposition (see e.g. Fig. 2): high-level categories can in fact be processed pre-attentively (and in a feed-forward manner) when stimuli are well separated, but this ability breaks down as soon as stimuli become too close to each other. Pre-attentive recognition abilities may well reflect the power – and limits – of the feed-forward sweep.
